# Relation of Growth Rate from Birth to Three Months and Four to Six Months to Body Mass Index at Ages Four to Six Years

**DOI:** 10.1155/2012/158643

**Published:** 2012-02-22

**Authors:** Robert J. Karp, Tawana Winkfield-Royster, Jeremy Weedon

**Affiliations:** Children's Hospital at Downstate, State University of New York (SUNY), Downstate Medical Center, P. O. Box 49, Brooklyn, NY 11203, USA

## Abstract

*Background*. While rapid early weight gain are common in children who become obese later in life, so is growth faltering in the first 3 months of life. *Objective*. We seek to determine what relationship weight gain in the first six months of age, separated into two 3-month periods, have with the BMI of children ages 4 to 6 years in an inner-city community. *Subjects*. A convenience sample cohort of 154 children attending an inner-city clinic. *Methods*. Consecutive charts were reviewed retrospectively. Age, gender, birth weight and weight change in the first and second 3 months of life were introduced as fixed factors using mixed linear models with BMI in years 4 to 6 as the dependent variable. *Results*. Weight change quartile in the first 3 months of life did not predict of BMI in years 4 to 6; however, weight changes quartiles during months 4 to 6 were significant predictors for subsequent overweight. *Conclusion*. The data presented herein suggest that, for this specific population, weight gain can be promoted when it is most essential. It is necessary, however, to identify intermediary variables that could affect outcomes in this and other communities.

## 1. Introduction

Growth failure in utero increases risk for both neurodevelopmental delays [1] and the elements of the Metabolic Syndrome (insulin resistance, coronary heart disease and hypertension) later in life [2, 3]. Paradoxically, continued growth failure limits impact of prenatal undernutrition on subsequent Metabolic Syndrome [4–6], but growth failure adds to the neurodevelopmental burden [7–9]. Thus absence of a prenatal: postnatal mismatch in nutrition diminishes risk for the health consequences and increases risk for developmental delay later in life in populations at risk for undernutrition [4–9].

 There have been dramatic increases in prevalence of pediatric obesity in the United States over the last 30 years [10–12]. School age children in impoverished African and Hispanic ancestry families are just as likely to be “overweight” or “obese,” defined as Body Mass Index (BMI) above the 85th percentile for age and gender, as they are to be lean [11, 13]. 

 The objectives for the present study are to determine what relationship, if any, (1) birth weight and (2) weight gain in the first six months of life, divided as first 3 months and second 3 months, have with BMI of children at 4 to 6 years of age. Variables available for study include birth weight, weights of infants at return visits to clinic in the first 6 months of life, and measures of weight and height with calculated BMI at 4 to 6 years of age.

## 2. Subjects and Methods

The subjects were a convenience sample cohort of 154 children attending the Resident Continuity Clinic at Suite D in Brooklyn, New York. Consecutive charts were reviewed retrospectively for the 154 children who had presented themselves for the set of immunizations commonly received at four years of age. The children ranged in age from four to six years.

The Suite D clinic serves an inner-city population in which approximately 31% of the children live in families with incomes below the poverty level [14]. The racial distribution of Suite D families is 81% African (“black”), 10% Hispanic, 5% European (“white—not Hispanic”), 1.5% Asian, and 2.5% other classifications [15]; however, the racial designations commonly used by the Census Bureau are not explanatory variables for this population, which is of predominantly Afro-Caribbean ancestry [16].

All children were born at the State University of New York-Downstate Medical Center. No charts were excluded. Multiple imputation of missing data was used to avoid low power and bias associated with complete set analyses [17]. SAS (SAS Institute, Cary, NC) PROC MI was used to impute 25 values from a normal distribution for each missing bodyweight value [18]. All data were obtained from patient records; no parental interviews were conducted.

Data points were added for all children over the subsequent 12 months, permitting assessment of BMI for children 4 to 6 years of age. Weight to the closest 10 grams and length at birth to 6 months of age and height at 4 to 6 years of age to the closest 0.5 cm were recorded with date of measurement using techniques described by Jelliffe [19].

Mixed linear models were constructed with BMI in years 4 to 6 as the dependent variable. Gender was introduced as a fixed factor as was age in years as use of a shorter age distribution would have increased the number of parameters in this complex model to the extent of unmanageability.

Following the postpartum age division made by Eid [20], birth weight and weight changes in the first 3 months and in the second 3 months of life were each divided into quartiles and added as factors. The presence of interactions among all of these factors was tested. A Handcock-Stein-Wallis structure was modeled for intrapatient covariance. Satterthwaite corrections to denominator degrees of freedom were applied. BMI scores were base-10 log-transformed to improve symmetry and homoscedasticity. Small numbers of outlying observations were excluded from analysis. Model-estimated BMI log-means with corresponding standard errors are reported. Software used was SAS Release 9.1.3 (SAS Institute, Cary, NC).

 The Suite D clinic serves a predominately Afro-Caribbean population that is representative of the community at large. Families served receive broad access, which is made available through public programs including Medicaid and State Child Health Insurance. These programs require that incomes be below 2.6 times the United States poverty level of $22,500 per year for a family of 4 people [14]. Of note, children in families that do not have documentation of legal status in the United States are eligible for these child insurance programs [19].

 Approval for the study was received from the Institutional Review Board of SUNY-Downstate Medical Center.

## 3. Results

Of the total of 699 observations of BMI during years 4 to 6 of life for the 154 patients, there were 366 observations belonging to 76 patients with weight recorded at both birth and 3 months; 280 observations belonging to 60 patients had weight recorded at birth, 3 and 6 months.

While girls were more likely to become overweight, gender was not a statistically significant predictor of BMI in years 4 to 6 of life (*F*[1,47] = 3.78, *P* = 0.058). Neither birth weight quartile (*F*[3,47] = 1.62, *P* = 0.197) nor weight gain quartile from birth to 3 months of age (*F*[3,47] = 0.26, *P* = 0.853) was a significant predictor of BMI in years 4 to 6 of life. Weight change quartile during months 4 to 6 of life, however, was a significant predictor of subsequent BMI at 4 to 6 years of life (*F*[3,47] = 3.47, *P* = 0.023).

Controlling for the other terms in the model, estimated log BMI (mean/standard error) in years 4 to 6 of life by weight gain category in months 4 to 6 is shown in Table 1. The means of the two lower quartiles differed significantly from those of the two upper quartiles. No statistically significant interactions among the predictors were detected.

The data presented herein show significant correlation between weight gain from four to six months of age and BMI at ages four to six years. These findings remained significant when adjusted for birth weight, age of measurement, and gender. By contrast, using the same data set, negligible and nonsignificant differences were found when comparing weight gain from birth to three months of age and BMI at ages four to six years; noneffect for weight gain measures below three months of age shown by *F* statistics were all less than 1.0.

## 4. Discussion

Prenatal experiences with nutritional deprivation have a profound impact on postnatal responses to abundance (Metabolic Syndrome) [1–6] or continued malnutrition (neurodevelopmental delay) [7–9]. Weight faltering, as described by Emond et al. in their review of the impact of infant growth on subsequent neurodevelopmental delay, is a common occurrence in this community [9].

The data presented herein provides evidence that weight gain in the first three months may not have the impact on subsequent overweight or obesity similar to that found for gain in the full first six months has. The data suggest that weight gain in the second three months following birth, however, is the significant predictor of Body Mass Index in years 4 to 6 in our population. The weight gain from birth to 3 months of age, however, did not contribute significantly to the prediction.

Findings for the full sample of 154 children, analyzed using techniques provided for disrupted series, remained robust when adjusted for birth weight, age of measurement, and gender (*F* test less than 1) [17].

Data from the present study showing weight gain for the first six months, considered in their entirety, are consistent with those of Eid [20], and of Dennison et al. [21] who observed that weight gain from birth to six months of life

“…was associated with increased risk of being overweight at 4 years of age; these findings were independently of potential confounders” [21].

Our data for weight gain in the first three months are, however, inconsistent with those provided by Stettler et al. in a multicenter study of 19,497 infants with a wide variety of social and ethnic backgrounds [22]. These authors used a cut-off of 4 months of age finding that “[a] pattern of rapid weight gain during the first 4 months of life was associated with an increased risk of overweight status at age 7 years” [22]. A microanalysis of their data, taken from a subset of 300 African American children from inner-city Philadelphia, replicated the correlation of rapid gain to 4 months of age and subsequent finding of overweight [23]. 

Limitations of the present study include using BMI as an outcome, as this measure does not necessarily imply obesity [12, 22, 23]. While children with BMI level at or above the 85th percentile are said to be overweight or “at risk” for obesity [12], the distribution of fat and muscle accumulation varies by age, ethnicity, timing of the “Adiposity (or “BMI”) rebound,” birth weight, and rate of weight gain [12, 24, 25]. Thus, accurate measures of fat deposition rather than lean muscle mass cannot be determined from a retrospective analysis of weight and height measures [24]. While data provided by Hediger et al. [24] suggest that the principle component of excessive weight gain in childhood is body fat, Wells et al. [25] provide data suggesting that effects of birth weight and rate of weight gain may influence lean and fat components of BMI differently in differing populations.

 An additional limitation is that our data are derived from measures of a population with the unique characteristics of central Brooklyn, NY, an inner-city borough of New York City with 2.3 million residents. There are 315,000 people in SUNY-Downstate's primary service area, the venue for the present study. The genetic, social, and economic influences on families attending Suite D are neither representative of the population of Brooklyn as a whole nor of other inner-city communities in the United States such as the one described by Stettler et al. [23]. This population is immigrant based from the Caribbean basin. The families live at or just above the poverty level which substantially influences parenting practices, food purchase and consumption patterns, and increases risk for obesity [12, 19, 26–30]. As Gopalan points out

“Differences…. between communities can result in important differences with nutritional status (especially of children) between households, and between communities with nearly similar overall levels of dietary inadequacy" [30].

Other variables likely to affect propensity for weight gain would include both paternal and maternal obesity [12], surgery to correct obesity [31], parental education [32], and food insecurity in the family [33]. The patient records reviewed to develop the data set for the current study do not have data to show differences between the population surveyed and others.

Use of data from the present study of a special sample in other populations and circumstances would require an in-depth analyses of antecedents, behaviors, and outcomes. A larger population would permit a shorter distribution of ages for outcome measures; however, observations drawn from macrosocial studies should not deter investigation of specific populations with unique characteristics, nor should data accumulated in microstudies be applied generally without careful investigation of applicability.

## 5. Conclusion

The data presented herein suggest that, for this specific population, weight gain can be promoted when it is most essential. Experiences in different populations are likely to differ. All require careful investigation of the variables affecting weight gain in the first months of life.

## Figures and Tables

**Table 1:** Mean log BMI at 4 to 6 years of age as a function of weight gain in months 4 to 6 of life. The data show that there are significant differences between means for quartiles 1 and 2 and means for quartiles 3 and 4.

Quartile	Gain range from 4 to 6 months of age (kg)	Mean log BMI at 4 to 6 years of age	Standard error
1	<1.5	1.200	0.09
2	1.5 to 1.9	1.201	0.08
3	1.9 to 2.5	1.229	0.09
4	>2.5	1.232	0.08
